# The Contribution of Sex, Personality Traits, Age of Onset and Disorder Duration to Behavioral Addictions

**DOI:** 10.3389/fpsyt.2018.00497

**Published:** 2018-10-16

**Authors:** Susana Valero-Solís, Roser Granero, Fernando Fernández-Aranda, Trevor Steward, Gemma Mestre-Bach, Núria Mallorquí-Bagué, Virginia Martín-Romera, Neus Aymamí, Mónica Gómez-Peña, Amparo del Pino-Gutiérrez, Marta Baño, Laura Moragas, José M. Menchón, Susana Jiménez-Murcia

**Affiliations:** ^1^Departament de Psicologia Clínica i de la Salut, Autonomous University of Barcelona, Barcelona, Spain; ^2^Ciber Fisiopatología Obesidad y Nutrición (CIBERObn), Instituto de Salud Carlos III, Madrid, Spain; ^3^Departament de Psicobiologia i Metodologia, Autonomous University of Barcelona, Barcelona, Spain; ^4^Pathological Gambling Unit, Department of Psychiatry, Bellvitge University Hospital-IDIBELL, Barcelona, Spain; ^5^Department of Clinical Sciences, Faculty of Medicine, University of Barcelona, Barcelona, Spain; ^6^Departamento de Educación y Psicología, Centro Universitario Cardenal Cisneros, Universidad de Alcalá, Madrid, Spain; ^7^Nursing Department of Public Health, Maternal and Child Health, University of Barcelona, Barcelona, Spain; ^8^CIBER Salud Mental (CIBERSAM), Instituto de Salud Carlos III, Madrid, Spain

**Keywords:** behavioral addictions, compulsive buying, internet gambling disorder, gambling disorder, sex addiction, age

## Abstract

**Background and aims:** Increases in the prevalence of behavioral addictions worldwide have led to a growth in the etiological research of the specific contribution of risk/protective factors to these disorders. The objective of this study was to assess the relative role of patients' sex, age of disorder onset and disorder duration on the clinical profile of behavioral addictions.

**Methods:** Our sample included treatment-seeking patients diagnosed with gambling disorder (GD, *n* = 3,174), internet gambling disorder (IGD, *n* = 45), compulsive buying (CB, *n* = 113), and sex addiction (SA, *n* = 34).

**Results:** The pattern of associations between the independent variables and the outcomes were strongly related to the behavioral addiction subtype: (a) for GD-men early onset of the disorder was related to GD severity, while for GD-women early onset was linked to novelty seeking; (b) for IGD-men, late onset correlated with addiction severity, worse psychopathological state, and high harm avoidance and self-transcendence levels; (c) for CB-women, early onset was related to higher reward-dependence scores and lower self-transcendence levels, and longer duration predicted higher cumulate debts; for CB-men, early onset and long duration correlated with high scores in harm-avoidance, self-directedness, self-transcendence, and cooperativeness; and (d) for SA-men, late onset and longer duration correlated with high disorder severity.

**Discussion and Conclusions:** These findings are relevant for developing prevention and treatment programs specific to different behavioral addictions.

## Introduction

Behavioral addictions include a heterogeneous group of conditions characterized by a compulsion to engage in a short-term rewarding, non-substance-related behavior that may engender persistence despite knowledge of severe adverse consequences (1–3). During the course of these problems, individuals lose control over excessive or problematic behaviors, with consequent significant impairment in the family, work and social areas of their lives (4, 5). In the early stages of the condition, high levels of impulsivity are aimed at obtaining immediate reward (positive reinforcement), but during the course of the condition, the addictive behavior becomes compulsive and is aimed at decreasing negative emotional states (negative reinforcement) (6). Within this line of research, it has also been argued that dimensional and transdiagnosis classifications could better explain the overlap of symptoms and shared clinical features in all these conditions, comorbidity, and even response to treatment (2).

The most prevalent subtypes of behavioral addictions are gambling disorder (GD), compulsive buying (CB), sex addiction (SA) and internet gaming disorder (IGD). In the latest version of the DSM-5, GD was included in the new diagnostic category named “Substance-related and Addictive Disorders,” while the possibility of including other behavioral addictions (such as CB, SA, and IGD) was discussed and excluded due to a lack of empirical evidence.

Behavioral addictions occur in people of both sexes, but prevalence differs depending on the subtype: men report higher percentages in GD, IGD, and SA, while women report higher rates of CB (7). In relation to age, these disorders occur throughout the life cycle, but two stages seem to have higher vulnerability: adolescence/ early adulthood and old age (8).

Studies exploring the contribution of the age of onset suggest that patients with earlier onset form a subgroup with higher levels of antisocial personality traits and impulsivity, whereas patients with later onset constitute a subtype with greater vulnerability to depression and anxiety, who use gambling as a maladaptative mechanism to modulate their negative moods (9, 10). Early onset of GD (compared to a later onset) also seems to be associated with a lower prevalence of mood disorders, a higher prevalence of cluster B personality disorders, higher scores in the personality trait sensation seeking and lower scores in self-directedness (11, 12).

Regarding CB, imprecise and unreliable prevalence results have been reported, ranging from 1 to 20% depending on the origin of the samples, definitions, and measurement instruments (13–17). Existing epidemiological data for CB have also shown that treatment-seeking patients with CB usually suffer from multiple psychiatric conditions, with comorbid alcohol and/or other drugs use, eating disorders, mood disorders, anxiety, and other impulse control disorders being most common (18). Strong sex-dependent differences for CB have been described: the risk, prevalence, and rates of initiation and frequency of misuse are higher for women (1).

Studies centered on IGD indicate that between 3.7 and 13.0% of the adult general population met criteria for problematic internet use (19, 20), and that IGD is more prevalent in young samples (21). Correlates of IGD include high levels of risk-taking behaviors and impulsivity, higher delay discounting, high sensitivity to social rejection and high levels in interpersonal conflict, harm-avoidance and interpersonal conflict (22–24).

Finally, research on SA concludes that prevalence for men is clearly higher compared to women (25–28). Higher socioeconomic levels, high scores in the personality traits sensation seeking and low scores in harm avoidance are risk factors for SA (29, 30). Some etiological studies have indicated that SA is related to antisocial personality traits, absence of fear, interpersonal assertiveness, egocentrism, and high levels in impulsiveness (31).

The fact that currently only GD is included in the DSM-5 derives from the lack of consensus regarding considering behavioral addictions as mental disorders (32). This could partly explain the higher prevalence of this disorder in relation to the other behavioral addictions. It is also challenging to determine the prevalence of conditions that are not accepted as disorders and do not dispose of standardized diagnostic tools (33). Having diagnostic criteria for these addictions would therefore allow for greater knowledge of the etiology, prevention and treatment of other behavioral addictions (34). Likewise, the inclusion of other behavioral addictions could have an impact on provided health services and might help to reduce the reluctance of patients to seek treatment (34).

### Objectives

To the best of our knowledge, a limited number of studies have measured the specific contribution of sex, onset and duration of addictive behavior in clinically heterogeneous samples including different behavioral addiction subtypes. Thus, the objective of this study was to assess the specific weight of these variables in the clinical state of treatment seeking patients diagnosed with GD, IGD, CB, and SA.

## Methods

### Participants

The sample included *n* = 3,366 consecutive treatment-seeking patients that attended a hospital unit specialized in behavioral addictions in Barcelona, Spain. Recruitment took place between January-2005 and Setember-2016. Inclusion criteria included meeting diagnostic criteria for GD, IGD, CB, or SA as the primary reason for consultation and being over 18 years of age. Exclusion criteria were having an intellectual disability or severe mental disorders (such as schizophrenia or other psychotic disorders or bipolar disorder).

The number of participants excluded due to the comorbid presence of different behavioral addictions was low (*n* = 5, 1 patient who reported GD+CB, 1 who presented GD+SA, 2 with CB+SA, and 1 with SA+IGD). On the other hand, since sub-samples of IGD and SA included very few women (n ≤ 2), female participants were excluded from these two groups to avoid potential biases in the results due the extremely low frequency of women in these two subsamples.

### Measures

#### Diagnostic questionnaire for pathological gambling according to DSM Criteria (35)

This 19-item questionnaire allows for the assessment of the DSM-5 (32) diagnostic criteria for GD. Convergent validity with the external gambling scores in the original version was very good (r = 0.77 for representative samples and r = 0.75 for gambling treatment groups; (35). Internal consistency in the Spanish adaptation used in this study was α = 0.81 for the general population and α = 0.77 for gambling treatment samples (36). In this study, the total number of DSM-5 criteria for GD was analyzed, and internal consistency was α = 0.804 in the sample.

#### Diagnostic criteria for compulsive buying (37)

These criteria, which have received wide acceptance in the research community, were used to validate the presence of CB in the sample. The list of questions explores “buying attitudes, associated feelings, underlying thoughts, and the extent of preoccupation with buying and shopping” (38).

#### Diagnostic criteria for IGD according to Griffiths and Hunt (39, 40)

To assess IGD diagnosis and to establish the level of dependence the disorder, clinical experts conducted a face-to-face interview considering the scale designed by Griffiths and Hunt (39, 40). This interview evaluated aspects such as the frequency of problematic behavior, the interference generated in daily functioning because of maladaptive use of internet games, the presence of tolerance and difficulties in abstinence management, as well as the number of DSM-5 criteria [according to Section III, (32)].

#### Diagnostic criteria for sex addiction according to DSM-IV-TR (32)

To assess SA, a battery of items was administered, which were based on the proposed definition in the DSM-IV-TR (41) in the Sexual Disorders Not Otherwise Specified section (302.9). In making our assessment, the following clinical description was given special weight: “distress about a pattern of repeated sexual relationship involving a succession of lovers who are experienced by the individual only as things to be used.”

#### Temperament and character inventory-revised (TCI-R) (42)

This is a reliable and valid 240-item questionnaire that measures seven personality dimensions: four temperament (novelty seeking, harm avoidance, reward dependence and persistence) and three character dimensions (self-directedness, cooperativeness, and self-transcendence). All items are measured on a 5-point Likert-type scale. A validated Spanish version was used (43). The scales in the Spanish revised version showed adequate internal consistency (Cronbach's alpha α mean value of 0.87). In the study, consistency indices ranged from good (α = 0.70 for novelty seeking subscale) to very good (α = 0.859 for persistence subscale).

#### Symptom checklist-revised (44)

This questionnaire evaluates a broad range of psychological problems and psychopathological symptoms. This questionnaire contains 90 items and measures nine primary symptom dimensions: somatization, obsession-compulsion, interpersonal sensitivity, depression, anxiety, hostility, phobic anxiety, paranoid ideation, and psychoticism. It also includes three global composite indices: (1) a global severity index (GSI), designed to measure overall psychological distress; (2) a positive symptom distress index (PSDI), to measure the intensity of the symptoms; and (3) a positive symptom total (PST), which reflects self-reported symptoms. A validated Spanish version was used (45). The Spanish validation scale obtained good psychometrical indexes, with a mean internal consistency of 0.75 (Cronbach's alpha). This study analyzes the GSI global score as a measure of the global psychopathological state (the consistency in our sample is excellent for this scale, α = 0.981).

#### Other sociodemographic and clinical variables

Additional demographic, clinical, and social/family variables were measured using a semi-structured face-to-face clinical interview described elsewhere (46). Covered variables included the age of disorder onset, the accumulated debts due to the addiction and social status measured through the Hollingshead index (a survey designed to measure social status of individuals based on educational attainment and occupational prestige; (47).

### Procedure

Experienced psychologists and psychiatrists, with more than 15 years of clinical experience in the field of addictive disorders, conducted two face-to-face clinical interviews in order to collect clinical information and specify the clinical diagnosis of each patient. All the measures analyzed in this study correspond to the assessment at the baseline, previous to the beginning of treatment.

### Statistical analysis

Statistical analysis was carried out with Stata 15 for Windows. Pearson's correlation coefficients measured the association between the age of onset and the duration of the problematic addictive behavior with the personality and clinical profile. The specific contribution of the patients' sex, onset, and duration of the problem on the severity of the addiction and the psychopathological state was measured with negative binomial regression and linear multiple regression (for cumulate debts and SCL-90-R GSI score). These models included and tested the interactions sex-by-onset and sex-by-duration: (a) for relevant interaction parameters, single effects for the participants' age were estimated into three groups defined for the quartiles 1 and 3 of the age of onset [early (onset before 20 years of age), medium (onset between 20 and 35 years) and late (onset after 35 years of age)]; and (b) for non-relevant interaction parameters, main effects were estimated and interpreted. Independent models were obtained for each diagnostic subtype (GD, CB, IGD, and SA). Contribution of sex was not explored for IGD and SA, since no women were included in these subsamples due to their low frequency in the groups.

### Ethics

This study was carried out in accordance with the latest version of the Declaration of Helsinki. The Ethics Committee of Bellvitge University Hospital (Barcelona, Spain) approved the study, and signed informed consent was obtained from all final participants.

## Results

### Characteristics of the sample

The upper half of Table 1 includes a description of the study sociodemographic variables. Mean chronological age for the total sample was 42.5 years-old (SD = 13.5, with a range between 18 and 75 years of age), mean age of onset for the behavioral addiction was 29.9 years-old (SD = 11.5) and the mean duration of the disorder was 6.2 years (SD = 5.9).

**Table 1:** Sample description: sociodemographic and clinical variables.

	**GD;** ***n*** = **3,174**		**IGD;** ***n*** = **45**		**CB;** ***n*** = **113**		**SA;** ***n*** = **34**			
	***n***	***%***	***n***	***%***	***n***	***%***	***n***	***%***	**χ^2^**	***p***
**GENDER**										
Females	283	8.9	0	0	85	75.2	0	0	502.6	<0.001
Males	2891	91.1	45	100	28	24.8	34	100		
**ORIGIN**										
Spain	2934	92.4	39	86.7	111	98.2	33	97.1	8.65	0.034
Immigrant	240	7.6	6	13.3	2	1.8	1	2.9		
**EDUCATION**										
Primary	1905	60.0	24	53.3	43	38.1	9	26.5	85.40	<0.001
Secondary	1092	34.4	20	44.4	46	40.7	16	47.1		
University	177	5.6	1	2.2	24	21.2	9	26.5		
**CIVIL STATUS**										
Single	1212	38.2	41	91.1	43	38.1	9	26.5	57.43	<0.001
Married - partner	1534	48.3	3	6.7	51	45.1	17	50.0		
Divorced - separated	428	13.5	1	2.2	19	16.8	8	23.5		
**SOCIAL INDEX**										
High	46	1.4	1	2.2	4	3.5	2	5.9	53.27	<0.001
Medium-high	138	4.3	0	0.0	18	15.9	4	11.8		
Medium	339	10.7	6	13.3	14	12.4	4	11.8		
Medium-low	967	30.5	12	26.7	32	28.3	14	41.2		
Low	1684	53.1	26	57.8	45	39.8	10	29.4		
**EMPLOYMENT**										
Unemployed	1414	44.5	36	80.0	53	46.9	15	44.1	22.69	<0.001
Employed	1760	55.5	9	20.0	60	53.1	19	55.9		
**^a^PREVIOUS CONSULTATIONS**										
No	374	11.8	2	4.4	13	11.5	2	5.9	3.43	0.330
Yes	2800	88.2	43	95.6	100	88.5	32	94.1		
	***Mean***	***SD***	***Mean***	***SD***	***Mean***	***SD***	***Mean***	***SD***	***F***	***P***
**AGE, ONSET AND DURATION**										
Age (years-old)	42.8	13.5	22.6	8.4	42.6	11.5	42.6	11.9	33.76	<0.001
Onset disorder (years-old)	29.9	11.5	19.3	8.1	32.9	12.0	33.7	13.0	16.82	<0.001
Duration disorder (yrs)	6.2	6.0	3.3	2.5	6.8	5.8	6.0	5.7	3.90	0.009
**PSYCHOLOGY: SCL-90R**										
GSI score	1.05	0.72	0.86	0.76	1.58	0.91	1.25	0.78	20.21	<0.001
**PERSONALITY TRAITS: TCI-R**										
Novelty seeking	108.9	14.3	103.7	13.1	114.9	14.4	110.8	14.3	7.85	<0.001
Harm avoidance	101.1	17.0	102.6	22.8	111.0	19.7	102.1	17.5	10.80	<0.001
Reward dependence	98.5	14.8	92.3	17.1	103.2	17.0	100.5	15.2	5.84	0.001
Persistence	108.5	20.1	93.6	20.8	106.8	18.8	103.6	21.1	8.20	<0.001
Self-directedness	127.0	21.1	127.1	25.7	124.1	23.9	116.9	19.6	2.91	0.033
Cooperativeness	130.4	16.3	126.8	18.5	133.9	15.7	127.4	15.1	2.57	0.053
Self-transcendence	64.0	15.3	57.2	14.1	65.4	16.5	63.1	14.0	2.97	0.031

*Note*.

a *Previous consultations due behavioral addictions related problems*.

*GD: gambling disorder. IGD: internet gaming disorder. CB: compulsive buying. SA: sex addiction*.

*SD: standard deviation. — This measure was not available for this group*.

The bottom half of Table 1 shows the distribution of the clinical variables and comparison between diagnostic subtypes. The IGD group included the youngest participants, with the lowest age on disorder onset and disorder duration. Regarding personality scores, CB endorsed as a whole the highest scores in novelty seeking, harm avoidance, reward dependence and self-transcendence, followed by GD.

### Associations between age of onset and duration with clinical and personality measures

Table 2 includes the correlation matrix to assess the association between the age of onset (years-old) and the duration (years) of each behavioral addiction with clinical measures. For the GD group, two associations emerged: in the male sub-sample, early onset was linked to a higher number of DSM-5 criteria, and in the female sub-sample, early onset was linked to higher novelty seeking scores.

**Table 2:** Association between age of onset and duration of the BA with clinical and personality traits.

	**GD**				**IGD**		**CB**				**SA**	
	**Women *n = 283***		**Men *n = 2,891***		**Men *n = 45***		**Women *n = 85***		**Men *n = 28***		**Men *n = 34***	
	**Onset**	**Durat**.	**Onset**	**Durat**.	**Onset**	**Durat**.	**Onset**	**Durat**.	**Onset**	**Durat**.	**Onset**	**Durat**.
DSM-5 total criteria	−**0.24**	0.10	−**0.19**	0.05	**0.44**	**−0.05**	–	–	–	–	–	–
Cumulate debts	**−0.14**	0.00	**−0.01**	0.04	–	–	**−0.15**	**0.25**	0.03	0.18	**−0.59**	**0.50**
***PSYCHOLOGY: SCL-90R***												
GSI score	**−0.11**	0.04	**−0.04**	0.10	**0.25**	0.11	**−0.03**	0.06	**0.36**	**−0.11**	**−0.18**	0.07
***PERSONALITY TRAITS: TCI-R***												
Novelty seeking	**−0.18**	0.15	**−0.25**	0.02	0.01	−0.05	**−0.19**	**−0.04**	**−0.05**	0.23	0.16	**−0.14**
Harm avoidance	−0.15	−0.06	0.07	0.07	**0.26**	0.08	−0.12	−0.12	**0.34**	−0.21	−0.08	0.07
Reward dependence	0.02	0.06	0.04	−0.07	−0.10	−0.11	**−0.27**	0.07	−0.07	0.17	0.17	−0.06
Persistence	−0.02	−0.06	−0.03	−0.07	−0.10	0.02	0.02	0.12	−0.01	**0.27**	−0.18	0.09
Self-directedness	0.06	−0.04	0.06	−0.09	−0.23	−0.15	0.07	0.09	**−0.25**	**0.25**	−0.03	0.14
Cooperativeness	0.01	0.00	0.09	−0.07	−0.13	−0.06	−0.05	0.13	**−0.28**	0.01	0.02	0.24
Self–transcendence	0.19	−0.03	0.16	0.05	**0.35**	0.22	**0.29**	0.08	0.19	**0.31**	0.01	0.08

*Note. GD, gambling disorder; IGD, internet gaming disorder; CB, compulsive buying; SA, sex addiction*.

*Note. Bold: correlation into the moderate (|r|>0.24) to good range (|r|>0.37). — Not available for this group*.

For women diagnosed with CB, younger age of onset was related to higher reward-dependence scores and lower self-transcendence levels, and the longer duration of the problem was associated with higher cumulate debts. For men in this diagnostic subtype (CB): (a) early age of onset was linked to lower SCL-90R scores and harm-avoidance levels, and high self-directedness and cooperativeness scores; (b) longer duration of the disorder correlated with higher levels in personality traits of persistence, self-directedness and self-transcendence.

For men who met criteria for SA, higher duration was related to higher hostility scores.

### Contribution of sex, age of onset, and duration to disorder severity

Table 3 contains the different regression models valuing the specific contribution of sex, age and duration of the disorder on behavioral addiction severity measures. Separate models have been obtained for each disorder (GD, CB, IGD, and SA) and for each severity measure (number of DSM-5 criteria, cumulate debs, and SCL-90R GSI score). For example, Model-1 assess the contribution of the independent variables of the study (sex, age and duration) on the dependent variable number of DSM-5 criteria specifically for the GD subsample. For each regression the non-standardized B-parameters, standard error (SE), 95% confidence interval (95%CI for B), contrast statistics (Wald-chisquare for negative-binomial regression and T for linear regression) and *p*-values are reported.

**Table 3:** Contribution of sex, onset and duration on the different BA subtypes.

	**B**	**SE**	**95%CI(B)**	**^c^Statistic**	***p***
**GAMBLING DISORDER;** ***n** = **3,174***						
**[Model-1] ^a^Criterion: DSM-5 criteria**
*(Intercept)*	*2.126*	*0.095*	*1.94*	*2.31*	*505.20*	*0.001*
Sex (0: women; 1:male)	−0.075	0.070	−0.21	0.06	1.16	0.282
Duration (years)	0.002	0.003	0.00	0.01	0.44	0.508
Age of onset (years-old)	−0.005	0.002	−0.01	0.00	9.18	**0.002**
**[Model-2] ^a^Criterion: cumulate debts**						
*(Intercept)*	*10.068*	*0.256*	*9.57*	*10.57*	*1543.67*	*0.001*
Duration (years)	0.012	0.004	0.01	0.02	10.58	**0.001**
^d^Sex (into early onset)	0.579	0.304	−0.02	1.18	3.63	0.057
^d^Sex (into medium onset)	0.323	0.106	0.12	0.53	9.29	**0.002**
^d^Sex (into late onset)	0.950	0.108	0.74	1.16	77.35	**<0.001**
Age of onset (into women)	−0.027	0.007	−0.04	−0.01	16.28	**<0.001**
Age of onset (into men)	0.023	0.002	0.02	0.03	106.92	**<0.001**
*Interaction: Sex by onset*	*0.050*	*0.007*	*0.04*	*0.06*	*50.62*	*0.001*
**[Model-3] ^b^Criterion: SCL-90R GSI**						
*(Intercept)*	*1.703*	*0.158*	*1.39*	*2.01*	*10.74*	*0.001*
Duration (years)	0.011	0.002	0.01	0.02	5.08	**<0.001**
^d^Sex (into early onset)	−0.433	0.163	−0.75	−0.11	−2.65	**0.008**
^d^Sex (into medium onset)	−0.634	0.074	−0.78	−0.49	−8.63	**<0.001**
^d^Sex (into late onset)	−0.422	0.073	−0.57	−0.28	−5.76	**<0.001**
Age of onset (into women)	−0.007	0.004	−0.02	0.00	−1.76	**0.048**
Age of onset (into men)	−0.002	0.001	0.00	0.00	−1.46	0.143
*Interaction: Sex by onset*	*0.005*	*0.004*	*0.00*	*0.01*	-*1.82*	*0.068*
**COMPULSIVE BUYING;** ***n** = **113***						
**[Model-4]** ^a^**Criterion: cumulate debts**						
*(Intercept)*	*11.149*	*0.437*	*10.29*	*12.00*	*652.20*	*0.001*
Sex (0: women; 1:male)	−0.497	0.246	−0.98	−0.01	4.07	**0.044**
Duration (years)	0.064	0.019	0.03	0.10	11.39	**0.001**
Age of onset (years-old)	−0.022	0.012	−0.05	−0.00	3.64	**0.050**
**[Model-5]** ^b^**Criterion: SCL-90R GSI**						
*(Intercept)*	*1.861*	*0.342*	*1.18*	*2.54*	*5.44*	*0.001*
Duration (years)	−0.010	0.016	−0.04	0.02	−0.66	0.508
^d^Sex (into early onset)	−0.430	0.572	−1.56	0.70	−0.75	0.453
^d^Sex (into medium onset)	−0.735	0.284	−1.30	−0.17	−2.59	**0.011**
^d^Sex (into late onset)	0.043	0.318	−0.59	0.67	0.13	0.893
Age of onset (into women)	−0.003	0.009	−0.02	0.01	−0.36	0.719
Age of onset (into men)	0.024	0.015	−0.01	0.05	1.61	0.112
*Interaction: Sex by onset*	−0.733	0.406	−1.54	0.07	−1.81	0.074
**INTERNET GAMBLING DISORDER;** ***n** = **45 (ONLY MEN)***						
**[Model-6] ^a^Criterion: DSM-5 criteria**						
*(Intercept)*	*0.752*	*0.540*	-*0.31*	*1.81*	*1.94*	*0.164*
Duration (years)	0.005	0.072	−0.14	0.15	0.01	0.940
Age of onset (years-old)	0.020	0.021	−0.02	0.06	0.93	0.335
**[Model-7] ^b^Criterion: SCL-90R GSI**						
*(Intercept)*	*0.231*	*0.372*	-*0.52*	*0.983*	*0.62*	*0.539*
Duration (years)	0.051	0.049	−0.05	0.150	1.05	0.298
Age of onset (years-old)	0.023	0.014	−0.01	0.052	1.63	0.110
**SEX ADDICTION;** ***n** = **34 (ONLY MEN)***						
**[Model-8] ^a^Criterion: cumulate debts**						
*(Intercept)*	*14.942*	*1.237*	*12.52*	*17.37*	*145.88*	*0.001*
Duration (years)	0.151	0.193	−0.23	0.53	0.62	0.432
Age of onset (years-old)	−0.259	0.045	−0.35	−0.17	32.84	**<0.001**
**[Model-9] ^b^Criterion: SCL-90R GSI**						
*(Intercept)*	1.651	0.449	0.74	2.57	3.68	0.001
Duration (years)	−0.005	0.025	−0.06	0.05	−0.18	0.856
Age of onset (years-old)	−0.011	0.011	−0.03	0.01	−1.01	0.321

a *Negative-binomial regression*.

b *Linear multiple regression*.

c *Wald-chisquare for negative-binomial regression and T for linear regression*.

d *Due to the relevant interaction sex by onset, single effects for the sex were obtained into three groups defined by the age of onset: early (onset before 20 years-old), medium (onset between 20 and 35 years-old), and late (onset after 35 years-old). Bold: significant predictor (0.05 level)*.

In the GD group, a higher number of DSM-5 criteria was associated to early age of onset (B = −0.005; *p* = *0.0*02), while no statistical contribution was obtained for the patients' sex or the duration of the disorder, and no interaction between sex and onset and duration was obtained for this criterion (Model-1 in Table 3). For this BA subtype, when the cumulate debts criterion is considered (Model-2 in Table 3) this measure is increased for patients with longer duration, and an interaction of sex by age of onset was also retained as relevant: (a) single effects for sex showed that men tended to cumulate more debts, and this difference increased with age of onset; and (b) single effects for age of onset showed that, for women, the earlier the onset, the higher the cumulate debts while for men, the older the onset, the higher the debts.

For the SCL-90-R GSI criterion (Model-3 in Table 3), worse psychopathological state was related to higher duration of the disorder, and the interaction sex by onset was also relevant to explain this outcome: (a) single effects for sex evidenced that women always registered higher psychopathological levels compared to men, but effect size for differences depended on the age of onset of the disorder (the highest effect was registered for onset between 20 and 35); and (b) the early age of onset of the disorder was only a statistical predictor of worse psychopathological state for women, while the onset of the disorder was not relevant in explaining GSI levels for men.

For the CB group, cumulate debts due to buying (Model-4 in Table 3) increased for women with a long duration and an early age of onset. No interaction between sex and age and duration emerged. The model adjusted for the global psychopathological level in CB (Model-5 in Table 3) retained the interaction sex by onset, and the results of this regression showed that worse mental state were registered for women compared to men, but only for patients who reported medium age of onset for the disorder (between 20 and 35 years old).

For the IGD and SA subsamples, sex was not included into the models since no women were included in these groups. For IGD, no statistical contribution of duration and onset emerged to explain the number of the DSM-5 criteria (Model-6 in Table 3) and the global psychopathological levels (Model-7 in Table 3). For men in the SA group, the cumulate debts increased for patients with younger age of onset (Model-8 in Table 3), while duration and onset were not contributors to psychological state (Model-9 in Table 3).

## Discussion and conclusions

This study assessed the association between patient sex, age of onset and behavioral addiction duration on clinical phenotype (including the severity of the disorder, psychopathological status and personality traits). Differences between GD, IGD, CB, and SA were tested, with the aim of shedding light on the potential moderating role of behavioral addiction subtype.

### Association of sex, onset and duration with severity, and psychopathological state

Our results indicate that, as a whole, the specific pattern of relationships was different depending on the behavioral addiction diagnosis. As a whole, our work provides new empirical evidence about the multidimensional component of behavioral addictions, for which the contribution of variables such as age of onset, duration of the disorder or personality traits play a differential role depending on the diagnostic subtype and the patients' sex. Previous studies had already found similar results regarding individual differences in behavioral addictions, noting that they must be conceptualized as a heterogeneous set of clinical conditions (1, 48).

The specific correlates of the age of onset also seem to be dependent on the diagnostic subtype and the patients' sex. This potential interaction is particularly notable for GD and CB: a) in GD, early onset correlated with greater severity in males and b) in CB, earlier onset correlated with higher levels of severity of the addictive problem in women (who presented higher prevalence of this diagnostic subtype compared to men, which is in accordance with previously reported clustering studies and latent profile analyses (18, 49).

### Association of sex, onset and duration with personality dimensions

The pattern of relationships between onset and duration of the behavioral addiction with personality is also different depending on the diagnostic subtype and the participants' sex. In GD, earlier age of onset has been associated with higher novelty seeking in men, which seems congruent with etiological research in male samples which consider age of onset as a mediating mechanism between novelty seeking levels with GD correlates (such as the disorder severity and psychopathology (11).

In CB, early onset was associated with high levels of reward dependence and low scores in self-transcendence in women, and with low scores in harm avoidance and high levels of self-directedness and cooperation in men. These results could indicate that women who met this condition could be more predisposed to seeking greater approval and to expressing more difficulties in coping with troubles or stressful situations, while men would be more goal-oriented, effective and self-confident. In addition, for the CB group, longer duration of the disorder was associated with higher persistence, self-directedness, and self-transcendence, which seems consistent with observations in previous studies (50, 51). Given our lack of females in the SA group, it is not possible to make associations between personality dimensions and sex in patients with this behavioral addiction. It would be beneficial for future studies to include more diverse and balanced samples. Still, we did identify that our SA sample endorsed higher levels of novelty seeking compared to GD patients, and importantly, low levels of self-directedness.

### Limitations and strengths

Despite having an overall large sample size (which provides a large statistical power for most analyzes and comparisons), the number of patients in some groups was relatively small. Likewise, due to the close relationship between the prevalence of each behavioral addiction and sex, the distribution of men and women was very unequal between the groups. It should be argued, however, that the sample included all patients who consecutively attended a reference treatment unit and met the inclusion/exclusion criteria, and therefore the gender distribution corresponds to the frequency with which these problems occur in our country (52), which gives high external validity to our results.

On the other hand, this work is aimed in knowing patients' sex, age, and duration of the disorder contribute *specifically* to the behavioral addiction which register the highest prevalence at a specialized healthcare unit in Spain, and therefore mutually exclusive groups have been analyzed. Future research must be designed to analyze the contribution of these variables on the clinical profile of patients who present concurrent-comorbid behavioral addictions.

Two strengths of our research are the large sample size and the inclusion of different groups of subjects who meet diagnostic criteria for different BA. Another relevant strength is the inclusion and analysis of multiple psychological measures, which cover severity of the BA, overall psychological state and personality traits.

### Implications

The results of this study provide new empirical evidence about the multidimensional component of behavioral addictions, for which the contribution of variables such as age of onset, duration of the disorder or personality traits should play a differential role depending on the diagnostic subtype and the patients' sex. Our results could be useful for future studies testing an integrative model for describing the underlying mechanisms which lead to the onset and development of each behavioral addiction diagnosis. As with most complex, multifaceted-multidimensional processes, further studies in different areas are needed: etiological research (for example neurological studies to recognize what specific regions, networks, and executive/cognitive functions are involved), and clinical studies (to identify the complete phenotypes and developmental trajectories of each diagnostic condition). Ultimately, a detailed understanding of the etiologically and the course of the behavioral addiction construct, as well as the underlying causes of its variability, will allow for improving prevention and treatment efforts. Special attention must be paid to the contribution of the socio-demographical features, and particularly to sex which seems have a complex contribution to the patients' clinical state depending on other external variables. Mental health preventive and intervention services will be benefit to undertake routine screening and assessment tools with high discriminative capacity for each diagnostic subtype, and to provide effective intervention programs that adequately manage the specific phenotypes. This is especially relevant for some types of behavioral addiction, for which there are few measuring tools and limited standard therapy plans exist (such as CB or SA).

## Author contributions

SV-S, RG, FF-A, JM, and SJ-M designed the experiment based on previous results and the clinical experience of NM-B, NA, MG-P, AdP-G, MB, and LM. SV-S, RG, VM-R, GM-B, TS, FF-A, and SJ-M conducted the experiment, analyzed the data, and wrote a first draft of the manuscript. SJ-M, TS, GM-B, RG, and FF-A further modified the manuscript.

### Conflict of interest statement

The authors declare that the research was conducted in the absence of any commercial or financial relationships that could be construed as a potential conflict of interest.
